# Probing the Action of Permeation Enhancers Sodium Cholate and N-dodecyl-β-D-maltoside in a Porcine Jejunal Mucosal Explant System

**DOI:** 10.3390/pharmaceutics10040172

**Published:** 2018-10-02

**Authors:** E. Michael Danielsen, Gert H. Hansen

**Affiliations:** Department of Cellular and Molecular Medicine, The Panum Institute, Faculty of Health Sciences, University of Copenhagen, 2200 Copenhagen, Denmark; gerth@sund.ku.dk

**Keywords:** intestinal permeation enhancers, sodium cholate (NaC), N-dodecyl-β-D-maltoside (DDM), small intestine, enterocyte, brush border

## Abstract

The small intestinal epithelium constitutes a major permeability barrier for the oral administration of therapeutic drugs with poor bioavailability, and permeation enhancers (PEs) are required to increase the paracellular and/or transcellular uptake of such drugs. Many PEs act as surfactants by perturbing cell membrane integrity and causing permeabilization by leakage or endocytosis. The aim of the present work was to study the action of sodium cholate (NaC) and N-dodecyl-β-D-maltoside (DDM), using a small intestinal mucosal explant system. At 2 mM, both NaC and DDM caused leakage into the enterocyte cytosol of the fluorescent probe Lucifer Yellow, but they also blocked the constitutive endocytotic pathway from the brush border. In addition, an increased paracellular passage of 3-kDa Texas Red Dextran into the lamina propria was observed. By electron microscopy, both PEs disrupted the hexagonal organization of microvilli of the brush border and led to the apical extrusion of vesicle-like and amorphous cell debris to the lumen. In conclusion, NaC and DDM acted in a multimodal way to increase the permeability of the jejunal epithelium both by paracellular and transcellular mechanisms. However, endocytosis, commonly thought to be an uptake mechanism that may be stimulated by PEs, was not involved in the transcellular process.

## 1. Introduction

The small intestinal brush border has two equally important, but mutually conflicting bodily functions to take care of: (1) to digest and absorb dietary nutrients, and (2) to prevent harmful luminal agents from gaining access to the body. Whereas the digestive/absorptive capacity of enterocytes is optimized by the large apical surface expansion represented by numerous microvilli [1,2], the unusually high glycolipid content of the apical membrane bilayer favors the formation of “lipid raft” domains [3,4,5]. The ensuing membrane robustness not only renders the brush border relatively resistant to noxious luminal agents, but also proves a challenge to overcome in the pharmacological quest for permeation enhancers (PEs) suitable for oral administration of drugs with otherwise poor bioavailability [6]. Surfactants represent a large and chemically diverse group of PEs, and as amphiphilic compounds, they have a capacity to adsorb to the cell membrane bilayer, thereby rendering it more fluid and causing it to expand, leading to a loss of integrity and an increase in permeability [6,7]. At higher concentrations, PEs may cause membrane solubilization, and ultimately, buckling and lysis of the cell membrane itself [6]; ideally however, PE action should strike the optimal balance between achieving adequate drug absorption from the gut without compromising mucosal integrity [8]. Functionally, intestinal PEs may act to increase transcellular pathways and/or the paracellular permeability by the opening of tight junctions between adjacent epithelial cells. Regarding the former, permeabilization is thought to be achieved by generating pores or leaks in the cell membrane, or by engaging one of several possible endocytotic mechanisms [6,9,10].

Bile salts are a distinct group of chemically related endogenous compounds engaged in assimilation of dietary fat [11]. Their role in this process may be likened to that of other types of surfactants, and they have long attracted considerable interest as potential PEs in drug absorption, as evidenced by their listing in several patents of oral peptide delivery systems [6,12,13,14]. Another class of widely-used soluble surfactant PEs is alkyl maltosides, of which members like bile salts are included in some formulations currently undergoing preclinical testing for oral drug delivery [6,15].

The aim of the present work was to compare how a bile salt, sodium cholate (NaC), and an alkyl maltoside, N-dodecyl-β-D-maltoside (DDM) affect the different cellular uptake pathways of a number of fluorescent polar and lipophilic probes, using a small intestinal mucosal organ culture model. This experimental setup is simple to use and offers a more in-vivo-like alternative to commonly-used epithelial cell lines such as Caco-2 [16]. Using this model, we have previously been able to visualize directly how other types of PEs, including lauroyl-carnitine, monocaprin, the ethoxylate C_12_E_9_, and melittin interact with the epithelium to affect uptake of polar and lipophilic probes [17,18,19]. At a concentration of 2 mM (~0.1%), NaC and DDM both exhibited multimodal actions as PEs, i.e., increased paracellular, as well as transcellular, permeability, without causing excessive damage to the integrity of the epithelium. At the ultrastructural level, NaC and DDM disrupted the normally ordered hexagonal organization of microvilli of the brush border and led to the extrusion of vesicle-like and amorphous cell debris to the lumen. Otherwise, overall cell morphology, including tight junctions and basolateral cell membranes, appeared intact, implying that the brush border is the primary target of both PEs. 

## 2. Materials and Methods

### 2.1. Materials

Sodium cholate hydrate (NaC, MW 409 Da) and N-dodecyl-β-D-maltoside (DDM, MW 511 Da) were supplied by Sigma-Aldrich (Copenhagen, Denmark; www.sigmaaldrich.com), Lucifer yellow CH ammonium salt (LY), a fixable analog of the FM lipophilic styryl dye FM 1-43 FX (FM), Texas Red Dextran (MW 3000 Da, lysine fixable) (TRD), ProLong antifade reagent with DAPI, and a monoclonal antibody to Na+/K+-ATPase (α-chain) by Thermo Scientific (Roskilde, Denmark; www.thermodanmark.dk), a rabbit antibody to intestinal alkaline phosphatase (IAP) was from AbD Serotec (Copenhagen, Denmark; www.bio-rad-antibodies.com), and a mouse anti-rat early endosome antigen-1 (EEA-1) antibody by BD Transduction Laboratories (Lyngby, Denmark; www.bdbiosciences.com). A rabbit antibody to pig intestinal aminopeptidase N (ApN) was prepared as previously described [20]. 

### 2.2. Animals

Animal experimentation included in this work was performed only by licensed staff at the Department of Experimental Medicine, the Panum Institute, University of Copenhagen, and was covered by license 2012-15-2934-00077 issued to the Dept. of Experimental Medicine, the Panum Institute, University of Copenhagen. A total of six animals were used in this study.

Segments of porcine jejunum, taken about 2 m from the pylorus of overnight fasted, postweaned animals, were surgically removed from the anaesthetized animals. After excision of the tissue, the animals were sacrificed by an injection with pentobarbital/lidocaine (1 mg/kg bodyweight). As this study contained no further animal experimentation, no specific approval by an ethics committee was required. 

### 2.3. Organ Culture of Porcine Jejunal Mucosa

Organ culture of small intestinal mucosa was performed essentially as described previously [16,21]. Briefly, after excision from the animals, jejunal segments of ~20 cm in length were quickly opened longitudinally and immersed in ice-cold RPMI medium. Mucosal tissue specimens weighing ~0.1 g were excised with a scalpel and placed on metal grids in organ culture dishes, to which 1 mL of pre-warmed RPMI medium was added. The dishes were placed in an incubator kept at 37 °C, and after 15 min of preincubation, reagents were added to the RPMI medium at the following concentrations: NaC and DDM (2 mM or 10 mM), LY (0.5 mg/mL), FM dye (10 µg/mL), and TRD (50 µg/mL). The explants were cultured in the presence of the reagents for 1 h in the dark, and after culture they were carefully rinsed in fresh RPMI medium and immersed in a fixative overnight at 4 °C. 

### 2.4. Fluorescence Microscopy

Explants were fixed at 4 °C overnight in 4% paraformaldehyde in 0.1 M sodium phosphate, pH 7.2 (buffer A). Following fixation and a quick rinse in buffer A, the explants were immersed overnight in 25% sucrose in buffer A before they were mounted in Tissue-Tek and sectioned (~7 µm thick sections cut parallel to the crypt-villus axis) at −19 °C in a Leica CM1850 cryostat. For immunolabeling, the sections were incubated for 1 h at room temperature with antibodies to EEA-1 (diluted 1: 100), Na^+^/K^+^-ATPase (diluted 1:100) or aminopeptidase N (diluted 1:5000) in 50 mM Tris-HCl, 150 mM NaCl, 0.5% ovalbumin, 0.1% gelatin, 0.2% teleostan gelatin, 0.05% Tween 20, 0.05% Triton X-100, pH 7.2 (buffer B). Incubation with Alexa-conjugated secondary antibodies (diluted 1:200 in buffer B) was for 1 h at room temperature. Controls with omission of primary antibodies were routinely included in the labeling experiments. 

All sections were finally mounted in antifade mounting medium containing DAPI, and examined in a Leica DM 4000B microscope (Leica, Wetzlar, Germany) fitted with a Leica DFC495 digital camera. Images were obtained using Leica HCX PL Fluotar objectives with the following magnification/numerical aperture: 20×/0.40, 40×/0.65, 63×/0.90, and 100×/1.30. The following filter cubes were used: I3 (band pass filter, excitation 450–490 nm), TX2 (band pass filter, excitation 560/40 nm), and A4 (band pass filter, excitation 360/40 nm). 

Hematoxylin-eosin (HE) staining of fixed tissue sections was performed according to a standard protocol.

### 2.5. Transmission Electron Microscopy

For transmission electron microscopy, cultured explants were fixed in 3% glutaraldehyde (*v*/*v*), 2% (*w*/*v*) paraformaldehyde in buffer A overnight at 4 °C. After a rinse in buffer A, the explants were post-fixed in 1% osmium tetroxide in buffer A for 1 h at 4 °C, dehydrated in acetone and finally embedded in Epon (an epoxy resin). Ultrathin sections were cut in a Pharmacia LKB Ultrotome III, and stained with 1% (*w*/*v*) uranyl acetate in H_2_O and lead citrate. All sections were finally examined in a Zeiss EM 900 electron microscope (Zeiss, Oberkochen, Germany) equipped with a Mega View II camera.

### 2.6. Preparation of Microvillus Membrane Vesicles and Treatment with NaC and DDM

Brush border microvillus membrane vesicles were prepared from pig jejunal mucosa by the divalent cation precipitation method [22]. Briefly, the mucosa was scraped from the gut and homogenized in 10 volumes of 2 mM Tris-HCl, 50 mM mannitol, pH 7.1, containing 10 µg/mL aprotinin and leupeptin. After centrifugation at 500× *g*, 5 min, MgCl_2_ was added to the supernatant (final concentration: 10 mM), and after 10 min on ice, the preparation was centrifuged at 1.500× *g*, 10 min. The resulting supernatant was collected and centrifuged at 48.000× *g*, 30 min, to yield a pellet of microvillus membrane vesicles (stored at −20 °C until use).

For subsequent treatments with NaC or DDM, microvillus vesicles (~1 mg/mL) were resuspended in 25 mM HEPES-HCl, 150 mM NaCl, pH 7.1, and samples of 75 µL were mixed with 25 µL of NaC or DDM to obtain a final concentration in the range of 0–10 mM. After 15 min incubation at 37 °C and rapid cooling on ice, the membrane suspensions were centrifuged at 20.000× g, 20 min, to yield a membrane pellet and a supernatant of solubilized protein. Both fractions were collected and subjected to SDS/PAGE in 10% gels, electrotransfered onto Immobilon membranes and stained for protein with Coomassie brilliant blue, as described in Section 2.8.

### 2.7. Detergent Resistant Membrane (DRM) Analysis of Microvillus Membranes by Sucrose Gradient Ultracentrifugation

One milliliter of microvillus membrane vesicles, prepared as described above, was resuspended in HEPES-buffer, pH 7.2. The membranes were then treated with NaC, DDM or Triton X-100 (all at a concentration of 1%) for 10 min on ice. A DRM analysis of the microvillus membranes was performed by sucrose density gradient ultracentrifugation by a method previously described [23,24]. Briefly, centrifugation was performed in a SW40 Ti rotor (Beckman Instruments, Palo Alto, CA) for 20–22 h at 35.000 rpm (*g_max_* = 217.000× *g*), and after centrifugation, the gradient was fractionated into a pellet and 12 fractions of 1 mL. Protein from each of the soluble fractions was isolated by acetone precipitation before analysis by SDS/PAGE. 

### 2.8. SDS/PAGE and Immunoblotting 

Samples were denatured by boiling for 3 min in the presence of 1% SDS and 10 mM dithiothreitol, and subjected to SDS/PAGE in 10% gels as described [25]. After electrophoresis and electrotransfer of proteins onto Immobilon PVDF membranes, immunoblotting was performed with antibodies to IAP (1: 1000 dilution), followed by horseradish peroxidase-coupled secondary antibodies (1: 2000 dilution). An electrochemiluminescence (ecl) reagent was used according to the manufacturers (GE Healthcare, www.gehealthcare.com) protocol to develop the blots. After immunoblotting, total protein was stained with Coomassie brilliant blue R250 (0.2% dissolved in an ethanol/H_2_O/acetic acid mixture (50:43:7).

## 3. Results

### 3.1. Microvillus Membrane-Solubilizing Effects of NaC and DDM 

**Figure 1:**
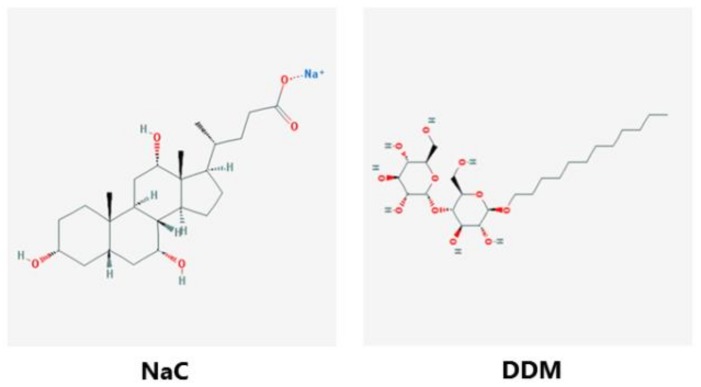
The chemical structure of NaC and DDM. (The images were downloaded from the PubMed Open Chemistry Data Base (https://www.ncbi.nlm.nih.gov/Structure/pdb/2MLT)).

**Figure 2:**
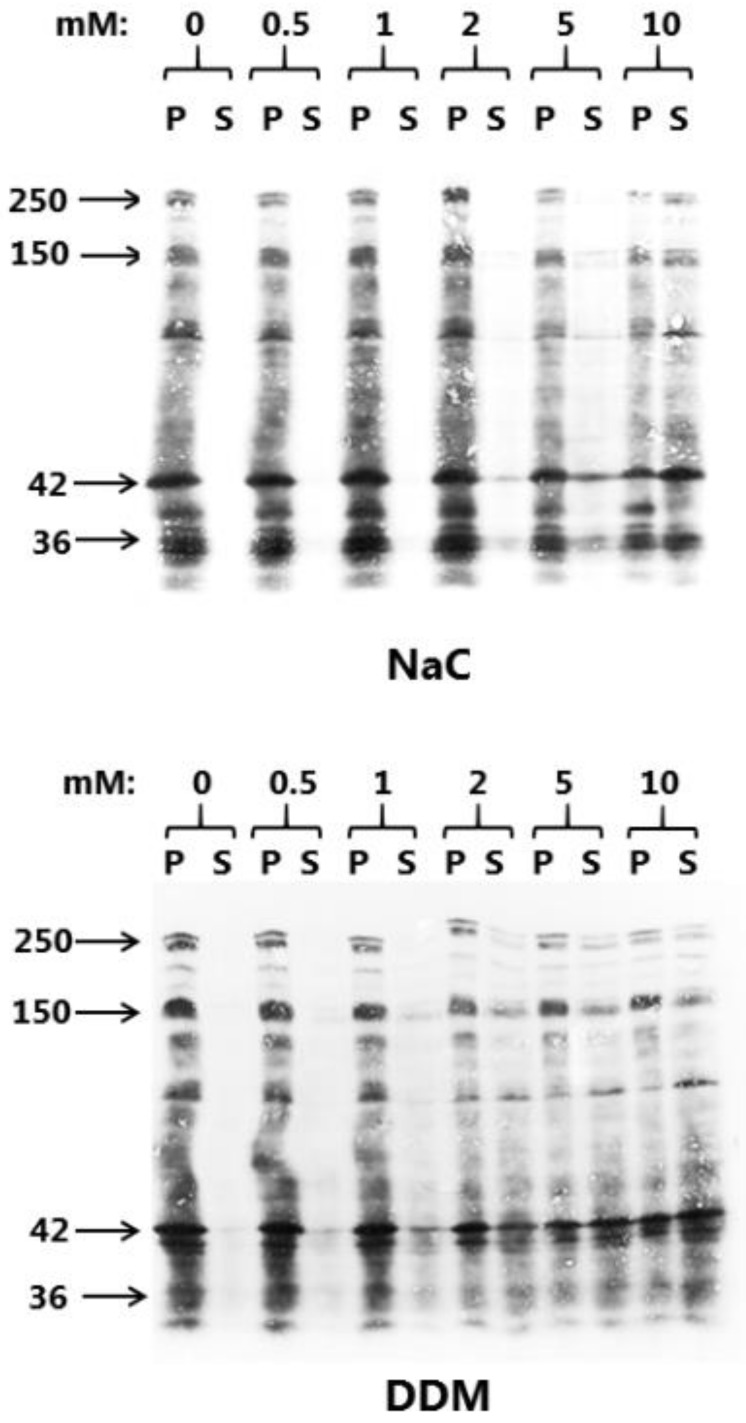
Solubilization of microvillus membrane vesicles with NaC or DDM at concentrations ranging from 0–10 mM as described in Methods. After centrifugation, the pellet (P) and supernatant (S) fractions were subjected to SDS/PAGE and proteins visualized by staining with Coomassie brilliant blue. Arrows indicate the molecular mass-values of the brush border proteins sucrase-isomaltase (250 kDa), ApN (150 kDa), actin (42 kDa), and annexin A2 (36 kDa).

Chemically, NaC and DDM belong to the large class of surfactant PEs of which the main common functional characteristic is the ability to adsorb to cell membranes, and thereby, to modify their physical and biological properties (Figure 1). In order to evaluate the membrane solubilizing effect on the intestinal brush border and to select optimal working concentrations of NaC and DDM for subsequent culture experiments, the concentration-dependency of their ability to release proteins from microvillus vesicles was first established. As shown in Figure 2, NaC had hardly any solubilizing effect above background levels at concentrations up to 2 mM, but at 5 and 10 mM, an increasing release of microvillus proteins was achieved. Both integral membrane proteins, such as the 150 kDa ApN, and the major microvillus cytoskeleton protein actin (42 kDa) appeared in the supernatant, indicating both membrane disruption and leakage. For DDM, microvillus solubilization was detectable already at 1 mM, consistent with the comparatively lower CMC-value for this PE (Figure 2). 

**Figure 3:**
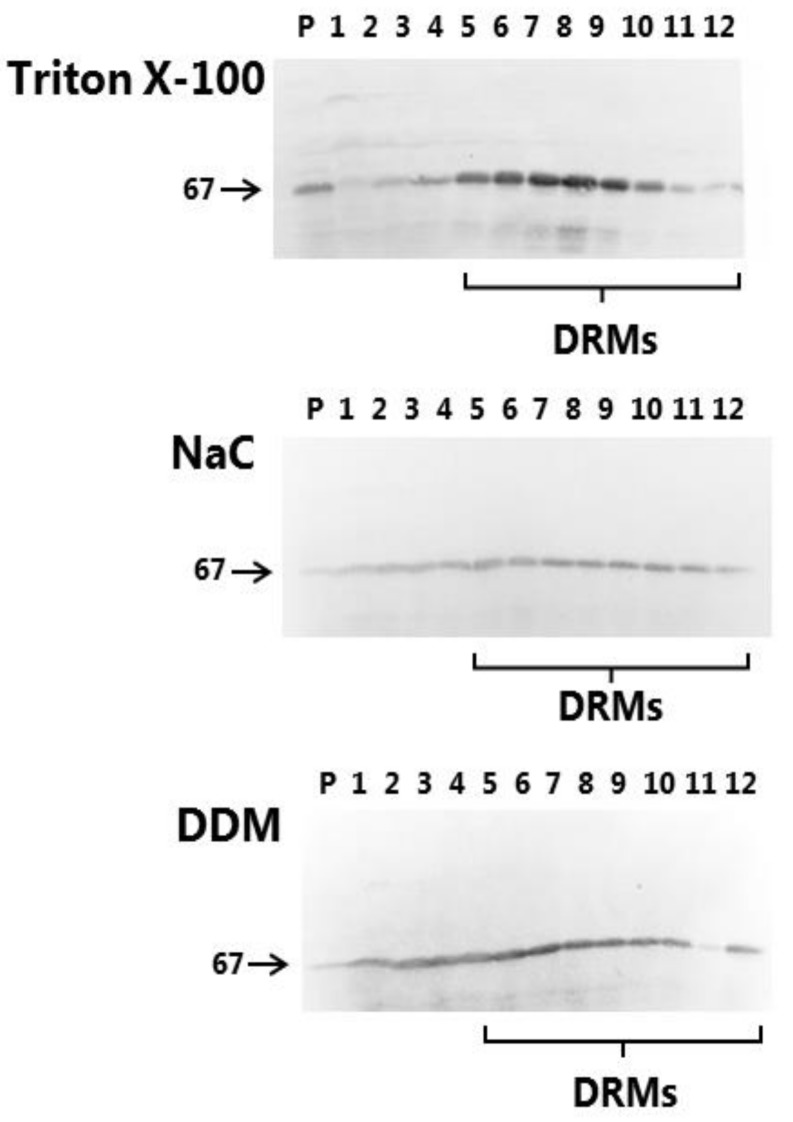
DRM analysis of microvillus membrane vesicles. Microvillus membrane vesicles were prepared and solubilized with either 1% Triton X-100, NaC or DDM on ice, followed by sucrose gradient ultracentrifugation, gradient fractionation, SDS/PAGE and immunoblotting for IAP (67 kDa). “P” indicates the pellet fraction, fractions 1-2 contain solubilized protein and fractions 4–12 contain DRMs.

The ability to resist solubilization by detergents/surfactants at low temperature is the biochemical hallmark of liquid-ordered membrane domains (i.e., lipid rafts) [5,26]. Thus, in the glycolipid-rich intestinal brush border, many of the major digestive hydrolases resist solubilization by the detergent Triton X-100, which is indicative that they reside in lipid rafts [24]. The 67-kDa IAP, linked to the extracellular leaflet of the membrane by a glycolipid anchor, is a well-known lipid raft marker [23], and as shown in Figure 3, this protein predominantly partitions in the floating DRM fractions of the sucrose gradient when using the “classical” detergent Triton X-100 for solubilization. An essentially similar DRM distribution for IAP was likewise observed for NaC, as well as for DDM, although relatively more of the enzyme appeared to be soluble. In addition, the profile of total microvillus protein also resembled that obtained with Triton X-100 (data not shown). This experiment thus indicates that the “non raft” domains of the brush border, i.e., those relatively enriched in phospholipids and poor in glycolipids, are those most vulnerable to the action of both PEs. 

### 3.2. Effects of NaC and DDM on Mucosal Morphology in Cultured Mucosal Explants 

Organ culture of mucosal explants is an ideal model system for investigating the direct, short-term effects of luminal compounds, and we have recently used it with a number of other types of permeation enhancers [17,18,19]. In subsequent mucosal organ culture experiments, NaC and DDM were both used at 2 and 10 mM in order to study cell-permeating effects under relative mild, as well as under more membrane damaging, conditions. Both concentrations (corresponding to 0.86% and 4.3% for NaC, and 1.0% and 5.1% for DDM, respectively) are well within the range of concentrations previously used by other investigators [6].

**Figure 4:**
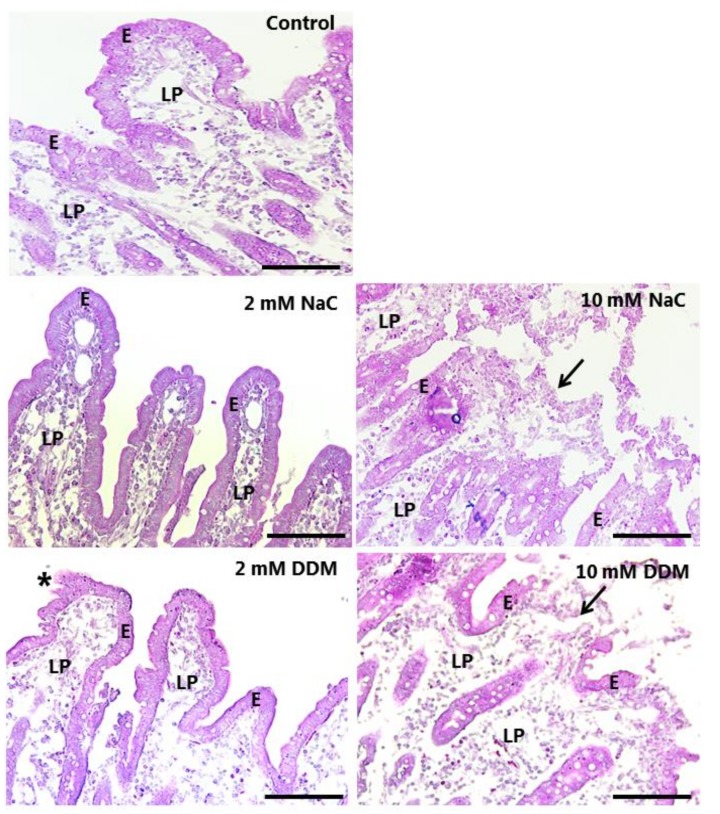
HE-stained sections of mucosal explants cultured for 1 h in the absence (control) or presence of 2 mM or 10 mM of NaC/DDM, as described in Methods. The villus organization and epithelium were generally well preserved at 2 mM of both surfactants, although foci of exfoliation was occasionally observed (asterisk). At 10 mM, both NaC and DDM caused extensive denudation, particularly near the tips of the villi (arrows), whereas the epithelium along the sides of the villi and in the crypts were generally better preserved. Enterocytes (E) and lamina propria (LP) are indicated. Bars: 100 µm.

As shown in Figure 4, exposure to either NaC or DDM at a concentration of 2 mM for 1 h caused no gross overall deterioration of the mucosal morphology. Villus height was preserved and the integrity of the epithelium generally upheld; only occasionally could foci of exfoliation be detected, typically at the tip of the villi. In contrast, at a concentration of 10 mM, both PEs caused extensive denudation at the tip of the villi, which are the most exposed and sensitive areas of the mucosal epithelium. Further down along the sides of the villi and in the generative crypts, the epithelium was generally well preserved. 

### 3.3. Effects of NaC and DDM on Permeability of Lucifer Yellow 

**Figure 5:**
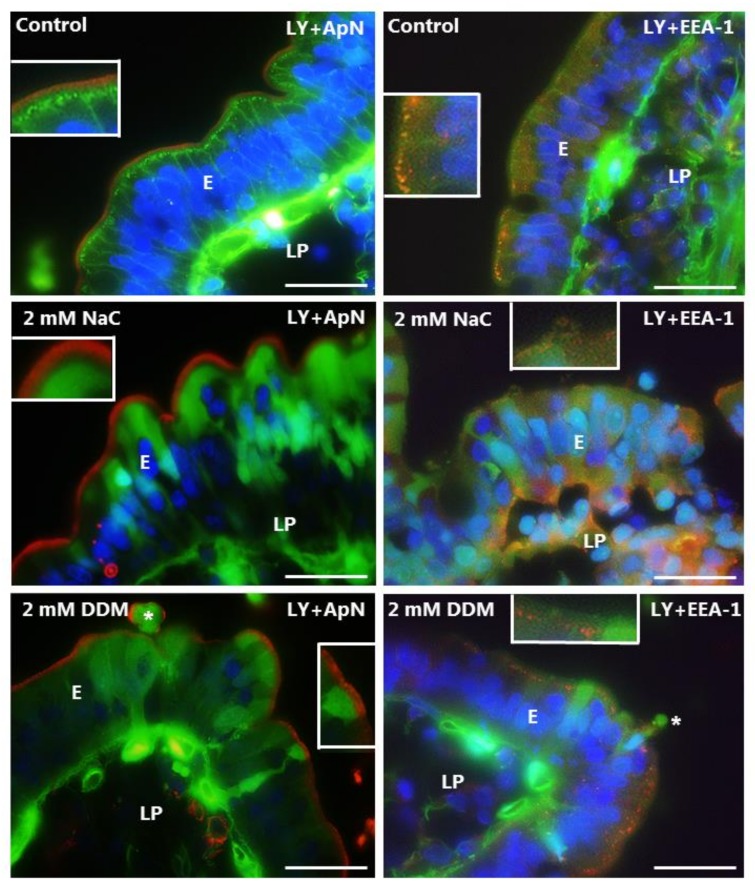
Permeability of the mucosal epithelium probed with LY at 2 mM concentration of PEs. Sections of mucosal explants cultured for 1 h with LY in the absence or presence of 2 mM NaC or DDM, respectively. In addition, the sections were immunolabeled for ApN, a brush border enzyme, or for EEA-1, a marker for early endosomes. In the controls, a string of EEA-1-positive subapical punctae shows uptake of LY into apical early endosomes of the enterocytes, whereas the cytosol is only weakly labeled. Lateral labeling and strong staining of the lamina propria are also seen, indicating a paracellular passage of LY through the tight junctions. At 2 mM, both NaC and DDM frequently caused LY to enter the enterocyte cytosol, indicating a leakage through the cell membrane, but endocytic uptake was not observed. Asterisks indicate enterocytes in the process of exfoliation. Inserts show enlarged image details. Bars: 20 µm.

LY is a small fluorescent polar tracer (MW 444 Da) with little or no permeability through normal cell membranes. It has previously been used to assess paracellular permeability in Caco-2 monolayers [27], and we have used it in the mucosal organ culture system for studying damage to epithelial cell integrity under various conditions [18,19,28]. As shown in Figure 5, little or no staining of the cytosol was seen in enterocytes of control explants, indicating well-preserved membrane integrity with little or no leakage through the cell membrane. Likewise, the brush border was devoid of staining by LY. However, an array of distinct subapical punctae was indicative of an uptake by constitutive endocytosis from the brush border. These punctae were EEA-1-positive, thus representing a subpopulation of early endosomes in the terminal web region of the enterocytes that we have previously termed “TWEEs” (Terminal Web Early Endosomes) [29]. The underlying lamina propria was strongly stained by LY in the control, and widespread stripy lateral staining along the enterocytes was indicative of a paracellular passage across the epithelium. Both NaC and DDM caused a diffuse staining of the enterocyte cytosol, indicating a leakage through the cell membrane. However, only some enterocytes appeared to be thus affected, resulting in a characteristic mosaic staining pattern of the epithelium; occasionally, single exfoliating cells were detected as well (Figure 5). The brush border marker ApN was confined to the apical surface in the presence of both PEs, indicating that no gross membrane disruption had occurred. However, noticeably, both NaC and DDM prevented the appearance of the LY-positive TWEEs, implying a blocked uptake via constitutive endocytosis. 

**Figure 6:**
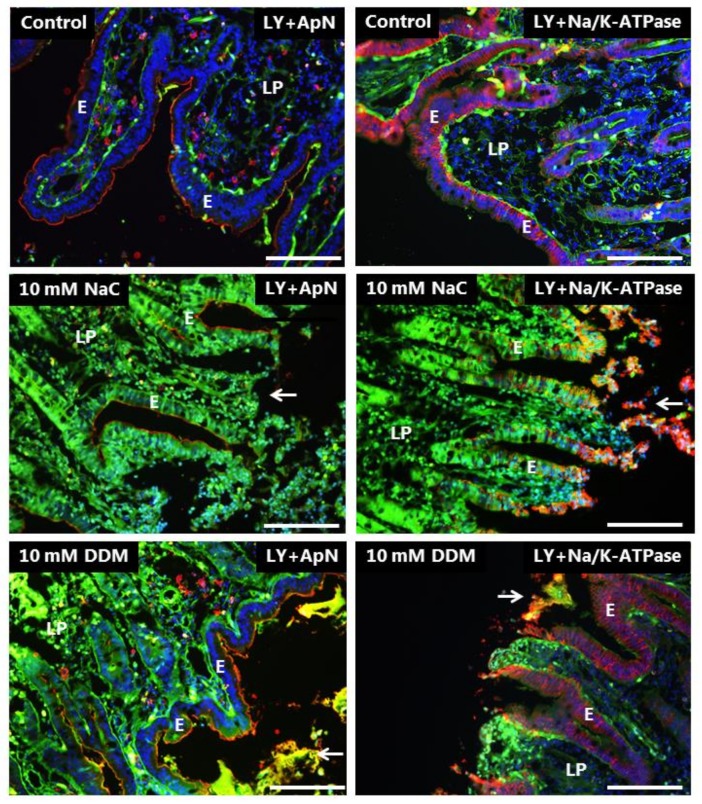
Permeability of the mucosal epithelium probed with LY at 10 mM concentration of PEs. Sections of mucosal explants cultured for 1 h with LY in the absence or presence of 10 mM NaC or DDM, respectively. In addition, the sections were immunolabeled for ApN, or Na^+^/K^+^-ATPase, a basolateral cell membrane marker. Both NaC and DDM caused extensive denudation at the tips of the villi (arrows), but epithelial integrity with preservation of cell membrane polarity was maintained along the sides of the villi. However, all enterocytes had taken up LY into the cytosol. Bars: 20 µm.

At a concentration of 10 mM, both NaC and DDM caused widespread leakage of LY into all enterocytes, although the localization of both the apical (ApN) -and basolateral marker (Na^+^/K^+^-ATPase) was seemingly unaffected in areas where the epithelium was still intact (Figure 6). 

### 3.4. Effects of NaC and DDM on Permeability of Texas Red Dextran and FM 1-43 FX 

**Figure 7:**
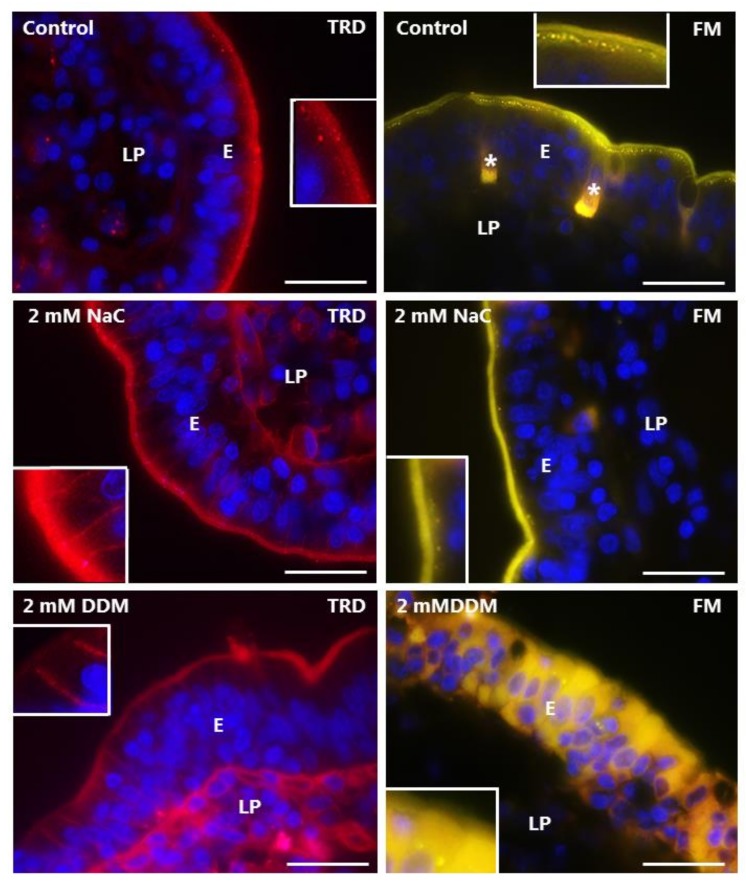
Permeability of the mucosal epithelium probed with TRD and FM. Sections of mucosal explants cultured for 1 h with TRD or FM in the absence or presence of 2 mM NaC or DDM, respectively. In the control, TRD bound to the enterocyte brush border, but only few and weakly-stained TRD-positive supapical punctae were observed. In addition, a faint labeling of the lamina propria was detectable. NaC and DDM both greatly increased the lateral TRD-labeling and accumulation of the probe in the lamina propria without affecting TRD-binding at the brush border, but little if any leakage was seen into the cytosol of the enterocytes. Like TRD, FM strongly labeled the enterocyte brush border, and also appeared in bright subapical punctae in the control. The brush border labeling was generally unaffected by both surfactants, but subapical punctae were sparse. Occasionally, FM was absent from the brush border, but leaked into the cytosol of enterocytes, as shown for DDM, but no staining of the lamina propria was observed. (Asterisks show goblet cells labeled by FM and inserts show enlarged image details.) Bars: 20 µm.

The TRD used in this study is polar like LY, but of higher MW (3000 Da), and is a commonly used probe for cellular uptake studies [30]. Unlike LY, it labeled the brush border of control enterocytes, but was only taken up sparsely into TWEEs in comparison with LY, implying a poor accessibility to the bottom part of the microvilli from where endocytosis occurs (Figure 7). TRD stained the lamina propria, but only weakly compared with LY, and a lateral staining along the enterocytes was barely visible, which was indicative of limited paracellular diffusion through the tight junctions. Binding to the brush border was unaffected by NaC and DDM, but no or little uptake into TWEEs was detected (Figure 7). However, both PEs greatly increased the staining of the lamina propria, and a stripy lateral labeling along the enterocytes was now clearly visible, implying a higher paracellular permeability of TRD. 

FM is a non-toxic, water-soluble and lipophilic probe (MW 560 Da) that only becomes fluorescent when incorporated into cell membranes [31], and as shown in Figure 7, it strongly labeled the entire brush border of control enterocytes together with goblet cells. In addition, it was efficiently taken up into TWEEs like LY, as earlier reported [29]. However, in contrast to LY, FM did not stain the lamina propria or the basolateral sides of the enterocytes, indicating that this lipophilic probe, despite its small size, is incapable of paracellular passage through the tight junctions. FM labeling of the brush border was generally not affected by NaC or DDM, but the uptake into TWEEs was greatly diminished (Figure 7). Lamina propria and lateral staining with FM was not detected in the presence of the PEs; however, as seen with LY, leakage into the cytosol of enterocytes was occasionally observed.

In summary, the results obtained with LY, TRD and FM documented both paracellular and transcellular mechanisms of action of both NaC and DDM. The data are consistent with the PEs interacting with/inserting into the brush border membrane, resulting in functional leaks in the bilayer that allow passage of both small polar (LY) and lipophilic (FM) compounds. In addition, the ability of the apical cell membrane to engage in endocytosis was largely abolished by both PEs. This effectively excludes the possibility that they act as PEs by stimulating luminal endocytotic uptake. 

### 3.5. Effects of NaC and DDM on the Ultrastructure of the Enterocyte Brush Border

**Figure 8:**
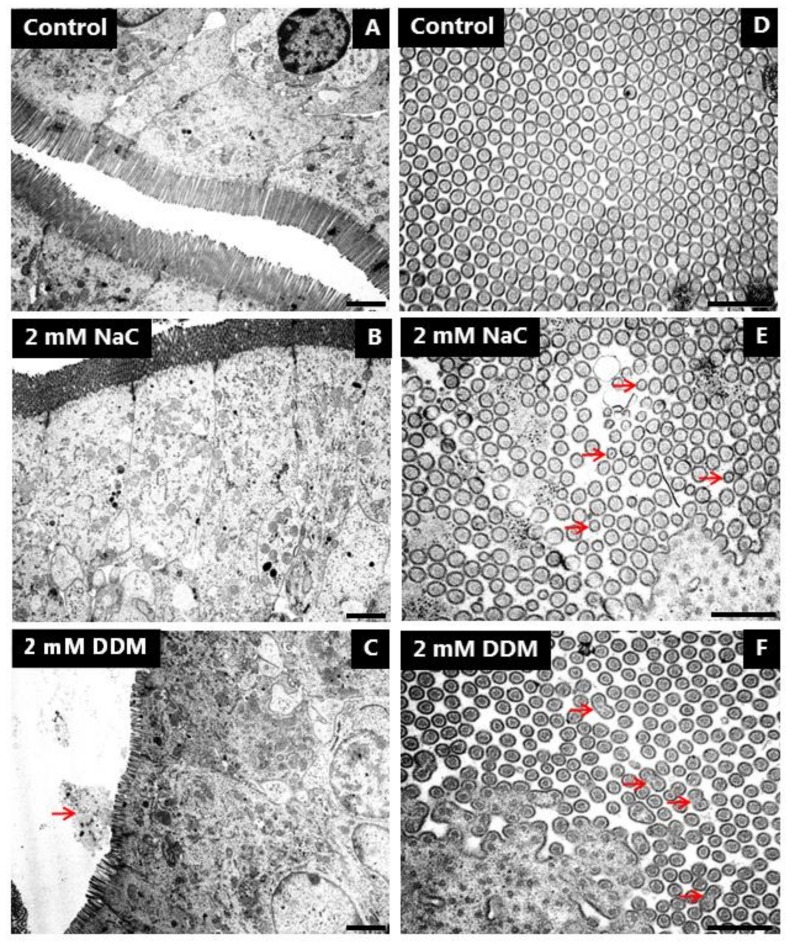
Effects of NaC and DDM on brush border ultrastructure. Electron micrographs showing longitudinal (**A**–**C**) and cross sections (**D**–**F**) of the brush border. (Arrows indicate apparent leakage into the lumen of cellular debris from the brush border in (**C**), microvilli with diminished diameter in (**E**) and fused microvilli in (**F**).) Bars: 1 µm (**A**–**C**); 0.5 µm (**D**–**F**).

**Figure 9:**
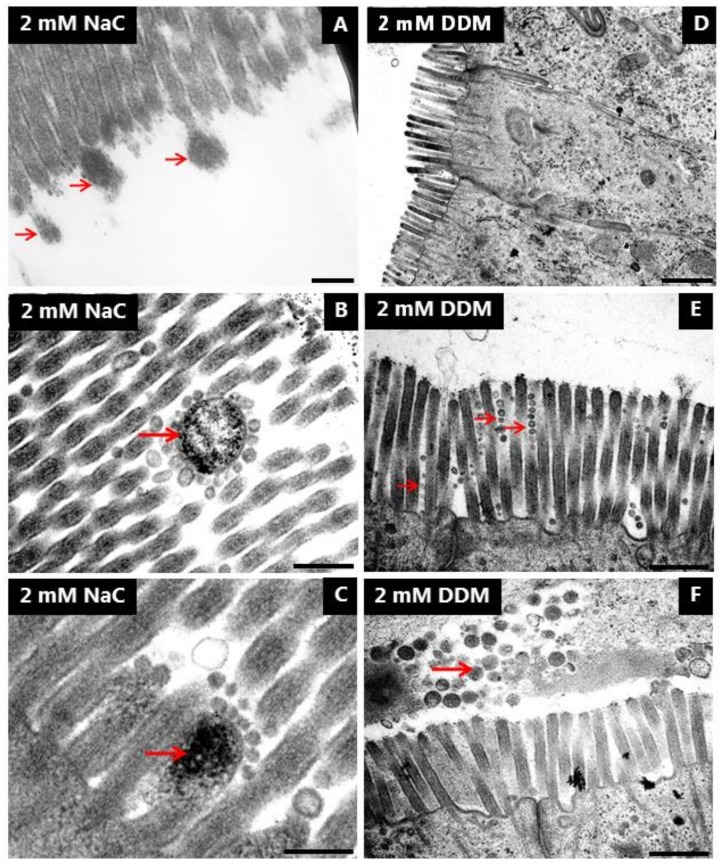
Effects of NaC and DDM on brush border ultrastructure. Electron micrographs showing different lesions in the brush border induced by NaC or DDM. Arrows indicate bulbous protrusions from single microvilli in (**A**), electron dense bodies with small vesicles attached in (**B**) and (**C**), microvesiculated, single microvilli in (**E**) and accumulated cellular debris in the lumen in (**F**). Bars: 0.2 µm (**A**–**C**); 1 µm (**D**); 0.5 µm (**E**,**F**).

Transmission electron microscopy was employed to study in closer detail the direct action of NaC and DDM on the morphology of the enterocytes. As shown in Figure 8A, the brush border of enterocytes from control explants in longitudinal sections from the mid-villus area consisted of a regular array of uniform microvilli of about 1.2–1.3 µm in length with actin rootlets extending into the subapical terminal web region. Microvillus length gradually increases in enterocytes along the villi as enterocytes move towards the extrusion zone at the tip, but microvillus diameter normally remains unchanged at ~0.1 µm throughout the crypt-villus migration, as shown in cross sections (Figure 8D). NaC (2 mM) did not affect the overall columnar shape of enterocytes or microvillus length in the brush border (Figure 8B). However, in cross sections, the normally ordered, hexagonal alignment of microvilli was less dense and frequently disrupted, and microvilli of smaller diameter were scattered amongst seemingly normal microvilli (Figure 8E). In addition, bulbous protrusions were occasionally observed at the tip of the microvilli (Figure 9A). Membrane-enveloped spherical bodies containing electron-dense material and with a diameter of about 0.3 µM were frequently observed interspaced between the microvilli, and in some cases they were surrounded by small (~50 nm) vesicles seemingly attached to their surface. (Figure 9B,C). 

Enterocytes were also generally well preserved after exposure to DDM (2 mM), but in contrast to NaC, DDM caused a marked shortening of the microvilli of villus enterocytes (Figure 8C). The effect was variable, but frequently cells with microvilli as short as 0.5 µm were observed. Although one must be cautious interpreting such data because, as mentioned above, microvillus length generally increases as enterocytes move along the crypt-villus axis, stunted microvilli like those seen with DDM are normally found only at the bottom of the crypts. In cross sections, the microvilli were of normal diameter, but as with NaC, the hexagonal alignment was less dense and organized than in the control. However, longitudinal fusion of pairs of microvilli was occasionally observed (Figure 8F). Noticeably, variation in microvillus length sometimes even occurred between neighboring cells (Figure 9D). At high magnification, microvesiculation of single microvilli was evident, and the tips of the microvilli often appeared flat instead of the normal rounded shape of a cap (Figure 9E). In areas near the bottom of the villi, vesicle-like structures and amorphous material, probably leaking through the brush border (Figure 8C), accumulated in the narrow cleft between opposing villi (Figure 9F). This phenomenon was observed both with NaC and DDM.

In contrast to the brush border, the tight junctions and meandering lateral cell membranes were seemingly unaffected by both PEs at 2 mM (data not shown). Taken together, the electron microscopy data therefore strongly indicate that the brush border is the primary target of both NaC and DDM. The lesions induced by NaC and DDM were similar, but not identical, implying that the two PEs cause disruption of the membrane organization by different mechanisms. 

## 4. Discussion

Surfactants belonging to the bile salt or alkyl maltoside classes of compounds all have a long history as PEs, but with regard to the intestinal permeation enhancement, they have so far been tested mainly either in epithelial cell models, most often Caco-2 cells, or in colonic tissue [6,32,33]. For this reason, we thought it worthwhile to reinvestigate the PE action of NaC and DDM in a jejunal mucosal explant system. The jejunum is the region of the intestine where endogeneous bile salts normally aid assimilation of poorly-soluble dietary constituents and where the brush border membrane presumably is optimally equipped to withstand the action of luminal surfactants under physiological conditions. 

In elucidating the PE action of a specific compound, it is essential to clarify the mechanism whereby epithelial permeabilization is achieved, i.e., whether a paracellular and/or transcellular route is facilitated [34,35]. Bile salts and alkyl maltosides are commonly classified as multimodal in their action, i.e., acting both via para and transcellular mechanism(s) [6,36], and the results of the present work agree well with this classification. Hypothetically, transcellular permeabilization may occur by endocytosis by one or more of several clathrin-dependent or independent molecular mechanisms known to internalize cell membrane and cargo to the cellular endosomal system. In enterocytes, apical endocytosis of cargo might be succeeded by trancytosis, leading to a complete crossing of the epithelial barrier [9,37,38]. In this context, the main conclusion of the present work is that neither NaC nor DDM achieved transcellular permeability by stimulating apical endocytotic uptake. In contrast, both largely abolished the constitutive endocytosis of polar (LY) and lipophilic (FM) probes that normally operates at the brush border and which can be readily visualized by these fluorescent compounds. Instead, transcellular permeabilization occurred by rendering the apical cell membrane leaky. The sometimes mosaic appearance of leaky enterocytes adjacent to unaffected cells together with the selective leakiness to the 444 Da LY, but not the 3-kDa TRD, implies that disruption of the membrane bilayer was not too extensive. This agrees well with the electron microscopy data, showing a reasonably well-preserved microvillus ultrastructure. Nevertheless, the partial break-up of the uniform hexagonal organization of microvilli and the appearance of scattered, abnormally thin (NaC) or fused (DDM) microvilli are direct visual evidence of PE-induced membrane damage. Some of the lesions observed in this study resemble those previously reported for intestinal brush borders of myosin-1a gene knockout mice, including disorganization of microvilli and bulbous protrusions from the microvillus tips [39]. Myosin-1a is a motor protein that links the microvillus actin cytoskeleton with the cell membrane and, amongst other functions, is responsible for the mechanical stabilization of the microvillus structure [1,2]. If PEs like NaC are able to disrupt this link, it could be the triggering event that ultimately leads to the observed morphological changes of the brush border. This suggests a stochastic process of disruption where single, affected microvilli form microvesicles and break up, followed by membrane resealing. Given the fact that the brush border architecture expands the apical surface area of the enterocyte roughly ten times compared to a “flat” cell surface [40], it harbors a large membrane reservoir for use in repair of damaged microvilli. Interestingly, microvillus microvesiculation in the rat and mouse has been proposed to be the result of a Ca^2+^-induced, villin-dependent severing of the actin filaments [41], and the subsequent release of vesicles into the lumen as a physiological process in the gut host defense aimed to deploy catalytic activities into the intestinal lumen [39,42,43]. Microvillus microvesiculation was very rarely observed in cultured control explants in this study on porcine jejunum, but the induction of this process by DDM may nevertheless reflect an uncontrolled triggering of a natural process. 

In recent studies, other types of surfactant PEs (lauroyl-carnitine, monocaprin and the ethoxylate C_12_E_9_) were found to permeabilize the brush border mainly by inducing microvillus microvesiculation and/or longitudinal fusion of microvilli [18], and the amphipathic cell penetrating peptide melittin caused a marked elongation both of microvilli and their actin rootlets [19]. Nevertheless, despite apparent differences in modes of microvillus disruption, these different types of PE agents all resulted in membrane leakage of LY as well as inhibition of apical endocytosis. Collectively, we infer from these observations that although the mechanism of action of the different types of membrane-interacting agents seems to vary, they possess a similar functionality as PEs. The common endocytosis-inhibiting effect may seem intriguing, and we are not certain of its cause. It could be ascribed simply to energy depletion by leakage of ATP or loss of essential other small molecules/ions, but local changes in membrane fluidity and lipid raft structure might also be sufficient to interfere with endocytosis even in areas where the microvillus architecture remains intact. The latter explanation is supported by the DRM analysis showing that “non raft” areas of the microvillus membrane are preferentially targeted by NaC and DDM. 

## 5. Conclusions

In the present work, we studied the action of a bile salt (NaC) and an alkyl maltoside (DDM) on the different luminal uptake pathways across the intestinal epithelium, using a jejunal mucosal organ culture model. Both PEs increased the paracellular and transcellular pathways for polar fluorescent probes, but of note, one common uptake mechanism, endocytosis, was virtually blocked. Instead, leakage through the brush border membrane occurred. Ultrastructurally, lesions of the brush border included microvillus microvesiculation, shortening, longitudinal fusion of microvilli, and the formation and extrusion of dense bodies. A DRM analysis of microvillus membrane vesicles indicated that both NaC and DDM, like the nonionic detergent Triton X-100, preferentially extract the “non-raft” domains of the lipid bilayer, thereby decreasing the fluidity of the remaining membrane.
